# Vaccination against connective tissue growth factor attenuates the development of renal fibrosis

**DOI:** 10.1038/s41598-022-15118-5

**Published:** 2022-06-29

**Authors:** Takashin Nakayama, Tatsuhiko Azegami, Kaori Hayashi, Akihito Hishikawa, Norifumi Yoshimoto, Ran Nakamichi, Erina Sugita, Hiroshi Itoh

**Affiliations:** 1 Department of Internal Medicine, Keio University School of Medicine, 35 Shinanomachi, Shinjuku-ku, Tokyo, 160-8582 Japan; 2 Keio University Health Center, 4-1-1 Hiyoshi, Kohoku-ku, Yokohama-shi, Kanagawa, 223-8521 Japan

**Keywords:** Kidney diseases, Peptide vaccines

## Abstract

There is a critical need for efficient treatment of chronic kidney disease (CKD). Renal fibrosis is a final common pathway to end-stage renal disease independent of the underlying etiology, and connective tissue growth factor (CTGF) is a well-recognized profibrotic factor in fibrosis of various organ systems. Here, we developed a novel peptide vaccine against CTGF to attenuate the development of renal fibrosis. Three inoculations with this CTGF vaccine at 2-week intervals elicited antibodies specifically binding to human full-length CTGF, and the antigen-specific serum IgG antibody titers were maintained for > 30 weeks. The efficacy of the CTGF vaccine on renal fibrosis was evaluated in adenine-induced CKD and unilateral ureteral obstruction (UUO) murine models. In adenine-induced CKD model, immunization with the CTGF vaccine attenuated renal interstitial fibrosis. Vaccinated mice showed low levels of serum creatinine and urea nitrogen and low urine albumin–creatinine ratio compared with vehicle-treated mice. In UUO model, the CTGF vaccination also suppressed the onset of renal fibrosis. In an in vitro study, CTGF vaccine-elicited IgG antibodies efficiently suppressed CTGF-induced- and transforming growth factor-β-induced α-smooth muscle actin expression in kidney fibroblasts. These results demonstrate that the CTGF vaccine is a promising strategy to attenuate the development of renal fibrosis.

## Introduction

Chronic kidney disease (CKD) is a global public health concern, affecting about 10% of the population worldwide^1^. There is a graded association between the severity of kidney dysfunction and the risks of death and cardiovascular events^2^. In 2017, CKD resulted in 1.2 million deaths and was the 12th most common cause of death worldwide^1^. Furthermore, it is predicted that death due to CKD will continue to increase up to 2060^3^. In addition to its negative effect on individual health, CKD also places a very serious economic burden on healthcare systems, and many patients with end-stage renal disease (ESRD) die without access to costly renal replacement therapy^4, 5^. Currently, about 2.6 million patients worldwide receive renal replacement therapy, and the numbers are expected to double by 2030^5^. Therefore, there is a high demand for the development of a new, more efficient strategy for the prevention of CKD progression to reduce the incidence of death, cardiovascular complications, and ESRD.

The only therapeutic options currently available for the prevention of CKD progression are blood pressure control and inhibition of the renin–angiotensin system^6, 7^. Because the pathogenic mechanism of development and progression of CKD is still obscure, specific treatment for CKD is not yet available. However, regardless of the underlying etiology, renal fibrosis is well known to be a final common pathway to ESRD and may be an attractive therapeutic target for CKD. Although the complicated mechanisms that promote renal fibrosis have not been fully elucidated, transforming growth factor-β (TGF-β) is conventionally regarded as the master regulator of fibrosis^8^. Previous studies have demonstrated that TGF-β promotes transformation of fibroblasts to myofibroblasts, resulting in excessive production of extracellular matrix and inhibition of its degradation^9, 10^. However, directly targeting TGF-β for antifibrotic therapy raises concerns because TGF-β has many crucial functions including the homeostasis of immune systems^8^ and the regulation of tumorigenesis^11^. Indeed, mutations in the TGF-β gene in mice have been associated with autoimmune disease^12, 13^. Therefore, prolonged and intensive inhibition of TGF-β is unlikely to be a viable therapy.

Connective tissue growth factor (CTGF), also known as CCN2 (cellular communication network factor 2), is one of the downstream profibrotic mediators of TGF-β activity^14^. Animal studies have demonstrated that lowering CTGF activity leads to decreased fibrosis in diverse fibroproliferative models^15–20^. In clinical studies, FG-3019, an anti-CTGF monoclonal antibody, reduced albuminuria in patients with diabetic kidney disease and slowed the rate of decline in forced vital capacity in patients with idiopathic pulmonary fibrosis with minimal adverse side effects^21, 22^. Based on the available evidence, inhibition of CTGF appears to be an attractive approach to attenuate renal fibrosis and accordingly slow the progression of CKD.

Because CKD often progress asymptomatically over time and therefore must be treated over the long term, medication adherence is an important consideration in the treatment of CKD^23^. Vaccination is well suited to the treatment of chronic diseases as well as the prevention of infectious diseases because it has the following characteristics: long-lasting effect, low frequency of administration, high level of compliance, and cost-effectiveness^24^. Therefore, vaccination may be a promising approach to prevent the progression of CKD.

In the current study, we developed a vaccine composed of the sequence of amino acids 150–156 of CTGF (common to both human and mouse) to attenuate the development of renal fibrosis, and then verified the efficacy of this vaccine in two renal fibrosis mouse models: adenine-induced CKD and unilateral ureteral obstruction (UUO) models.

## Results

### Development of specific antibodies against CTGF

CTGF consists of four distinct conserved domains. Among these domains, domain 2 plays an important role in fibrogenic signaling through the interaction with TGF-β and bone morphogenic proteins (BMPs), which suggests this domain is an appropriate target for a vaccine^25^. In addition, to increase the potential of neutralizing antibody production the three-dimensional structure, hydrophilicity, and electric charge also need to be considered, and furthermore, it is preferable that the epitope of the vaccine be as short as possible to avoid a harmful T cell response^26^. Taking into account these factors, we finally selected the sequence of amino acids 150–156 in domain 2, a sequence which is identical in mice and humans, as the epitope for the vaccine.

**Figure 1:**
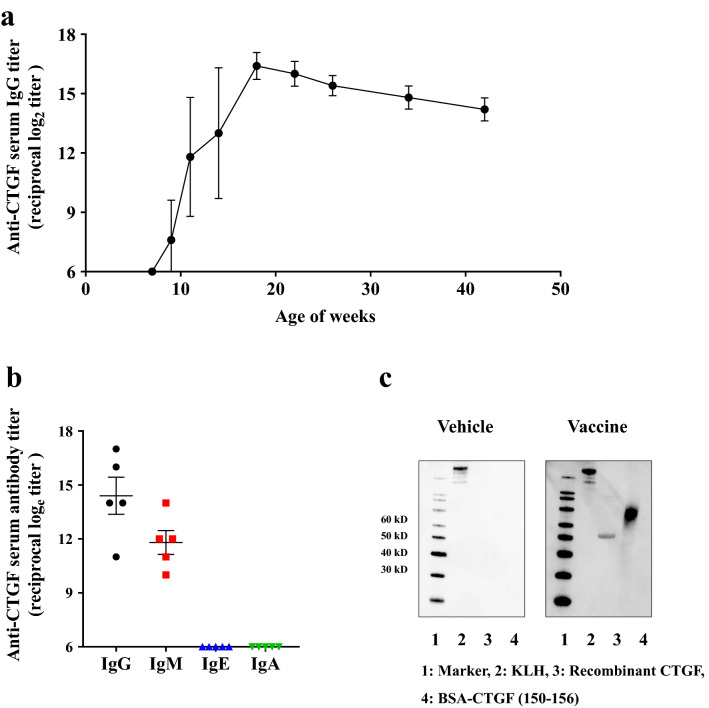
CTGF vaccination elicits antigen-specific antibodies in mice. Male C57BL/6 J mice were inoculated with the CTGF vaccine at 6, 8, and 10 weeks of age, and serum antigen-specific antibody titers were determined by using ELISA (*n* = 5). (**a**) Variation in serum IgG antibodies against the antigen was examined from 7 to 42 weeks of age. The antibody titer rapidly increased after the third inoculation, reaching a peak at 18 weeks of age. After that, it was maintained at a relatively high level until 32 weeks after the third inoculation. (**b**) Serum immunoglobulin isotypes of antibodies against the antigen in mice immunized with the CTGF vaccine were evaluated at 11 weeks of age. IgG antibodies were dominant although IgM antibodies were also detected at a certain level. IgE and IgA antibodies were scarce. (**c**) Western blotting analyses showed that serum from mice immunized with the CTGF vaccine specifically bound to not only the vaccine antigen but also to rhCTGF. In contrast, serum from mice inoculated with the KLH vehicle did not demonstrate binding to CTGF vaccine antigen or rhCTGF. Error bars show mean ± SEM.

The CTGF partial peptide was conjugated to the carrier protein keyhole limpet hemocyanin (KLH) to enhance the immunogenicity and stability, and 20 µg of the CTGF–KLH peptide was subcutaneously administered to male C57BL/6 J mice a total of three times (at 6, 8, and 10 weeks of age) in combination with Freund’s adjuvant (*n* = 5). The serum antibody titers against the epitope antigen were determined by using enzyme-linked immunosorbent assays (ELISA). The antigen-specific serum IgG antibody titers remarkably increased after the third inoculation and achieved a peak at 18 weeks of age (reciprocal log_2_ titer 16.2 ± 0.6) (Fig. 1a). After that, the IgG antibody titers were maintained at high level until 42 weeks of age, when the experiment was terminated (reciprocal log_2_ titer 14.2 ± 0.6) (Fig. 1a). Regarding the other classes of immunoglobulin, as of 1 week after the third inoculation, IgM antibody titers were detected in a lower level compared with IgG antibody titers, whereas IgA and IgE antibodies were scarce (Fig. 1b). Subsequently, the vaccine-elicited antibodies were evaluated for their ability to bind to recombinant human full-length CTGF (rhCTGF). Western blotting analyses showed that antibodies present in the serum of mice inoculated with the vaccine, but not the vehicle, recognized rhCTGF as well as the epitope antigen (Fig. 1c). These results indicated that the CTGF vaccine efficiently elicited antibodies specifically binding to CTGF.

### Effects of the CTGF vaccine in the adenine-induced CKD model

**Figure 2:**
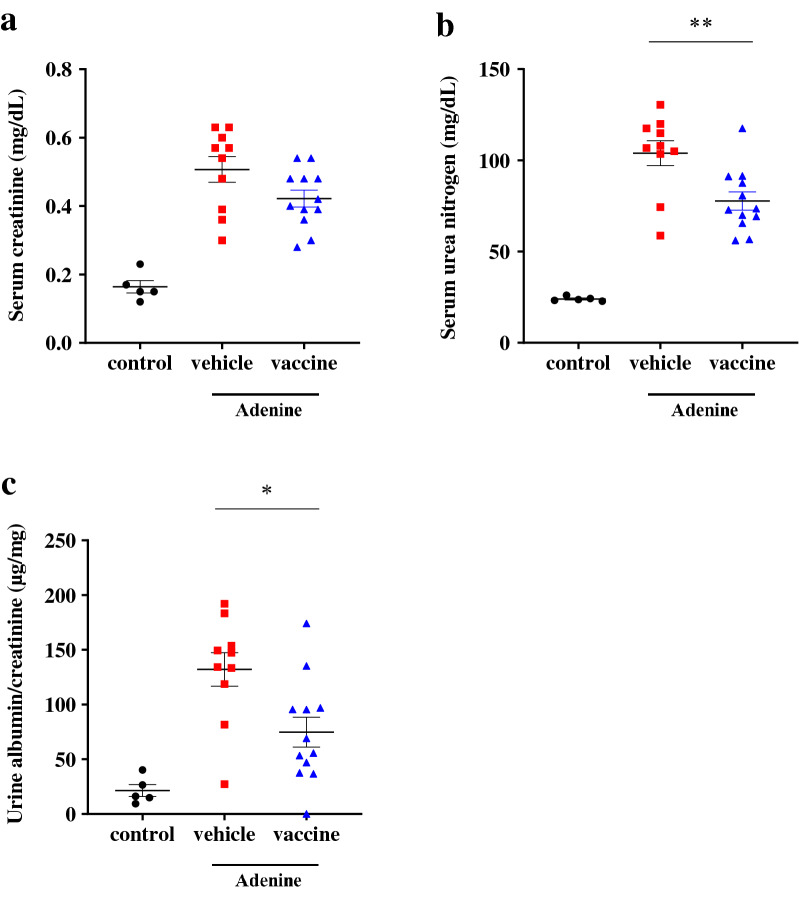
Effects of the CTGF vaccine on renal functions in the adenine-induced CKD model. Male C57BL/6 J mice were inoculated with the KLH vehicle or CTGF vaccine. Blood and urinary samples were collected after feeding the mice a rodent diet containing 0.2% adenine for 3 weeks (*n* = 10–12/group). (**a**) Serum creatinine and (**b**) urea nitrogen levels were measured, and (**c**) urine albumin/creatinine ratios were calculated. Error bars show mean ± SEM. Statistical difference between the KLH vehicle and CTGF vaccine group was determined by using Student’s *t*-test. **P < 0.01; *P < 0.05 as compared with the vehicle.

**Figure 3:**
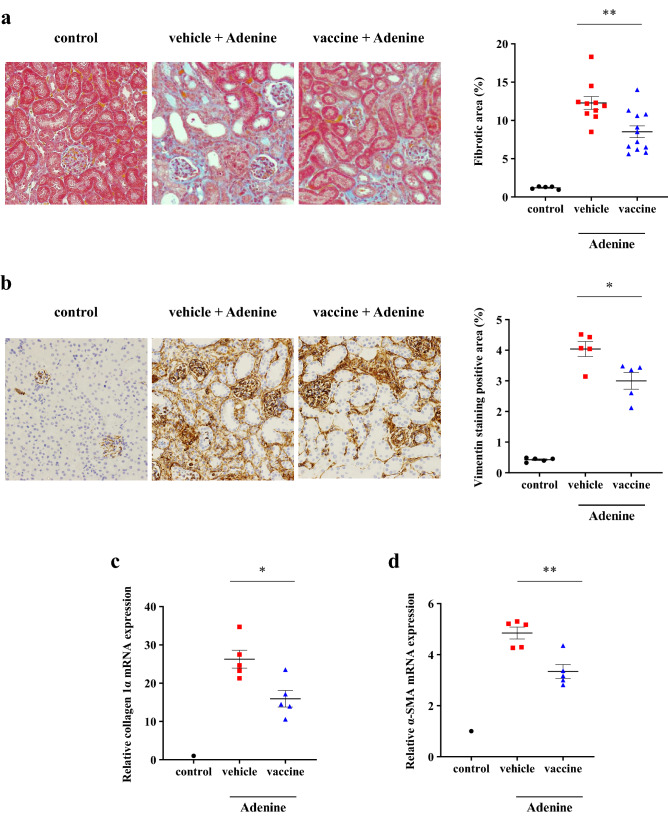
Effects of the CTGF vaccine on renal interstitial fibrosis in the adenine-induced CKD model. Male C57BL/6 J mice were inoculated with the KLH vehicle or CTGF vaccine. Mice were euthanized after feeding a rodent diet containing 0.2% adenine for 3 weeks (*n* = 10–12/group). Kidney Sects. (4 µm) were stained with (**a**) Masson’s trichrome and (**b**) vimentin. Ten unselected fields per mouse were captured at × 200 magnification, and stained areas were quantified as a percentage of the total area. The relative mRNA expressions of (**c**) collagen 1α and (**d**) α-SMA normalized to β-actin were measured with real-time PCR (*n* = 5/group). Error bars show mean ± SEM. Statistical difference between the KLH vehicle and CTGF vaccine group was determined by using Student’s *t*-test. **P < 0.01 as compared with the vehicle.

To investigate the effects of the CTGF vaccine on renal fibrosis, we used an adenine-induced CKD model: excessive adenine is metabolized to 2,8-dihydroxyadenine, which precipitates in the tubules resulting in macrophage infiltration and tubulointerstitial fibrosis^27^. Mice were divided into the non-treated control, KLH vehicle, or CTGF vaccine group at 6 weeks of age (*n* = 5, 12, and 12/group, respectively). From 12 weeks of age (2 weeks after the last inoculation with the vaccine or vehicle), mice in the vehicle and vaccine groups were fed a diet containing 0.2% adenine for 3 weeks. Two mice in the vehicle group were found dead before the planned end point (at 14 and 15 weeks of age, respectively); these mice were excluded from the analyses. Metabolic parameters are shown in Table 1. There was no significant difference in food or water intake between the vaccine and vehicle group, whereas body weight at 15 weeks of age was significantly higher, and urine output tended to be lower in the mice inoculated with the vaccine compared with the vehicle-treated mice. Regarding renal function, adenine feeding caused elevated serum urea nitrogen (UN) and serum creatinine levels (Fig. 2a, b). However, elevated UN level was significantly lower and elevated serum creatinine level tended to be lower in the vaccine group compared with the vehicle group (UN: 77.7 ± 5.0 vs 104.0 ± 6.8 mg/dL; *P* < 0.01. Creatinine: 0.42 ± 0.02 vs 0.51 ± 0.04 mg/dL; *P* = 0.06) (Fig. 2a,b). Vaccination also significantly reduced increased urine albumin/creatinine ratio (74.7 ± 13.8 vs 132.0 ± 15.3 µg/mg; *P* < 0.05) (Fig. 2c). Histopathological analyses using Masson’s trichrome-staining showed that adenine feeding caused the expansion of the interstitial fibrotic areas (Fig. 3a). Increased fibrotic areas were significantly attenuated in the vaccine group compared with the vehicle group (8.5 ± 0.8% vs 12.3 ± 0.8%; *P* < 0.01) (Fig. 3a). The increased area of vimentin positive staining was also significantly reduced in the vaccine group compared with the vehicle group (3.0 ± 0.6% vs 4.0 ± 0.5%; *P* < 0.05) (Fig. 3b). The increased mRNA expressions of collagen 1α and α-SMA in kidney tissue samples were significantly suppressed in the vaccine group compared with the vehicle group (*P* < 0.05 and *P* < 0.05, respectively) (Fig. 3c,d). These data indicated that the CTGF vaccine attenuated the development of renal interstitial fibrosis and kidney dysfunction.

**Table 1:** Metabolic parameters.

Variables	Control (*n* = 5)	Vehicle (*n* = 10)	Vaccine (*n* = 12)
Weight at 12 weeks of age (g)	25.5 ± 0.4	25.3 ± 0.2	25.6 ± 0.4
Weight at 15 weeks of age (g)	27.2 ± 0.7	21.0 ± 0.4	22.5 ± 0.5*
Food intake (g/day)	3.5 ± 0.4	2.5 ± 0.3	2.8 ± 0.2
Water intake (mL/day)	4.3 ± 0.5	9.0 ± 0.9	9.8 ± 0.7
Urinary volume (mL/day)	0.8 ± 0.2	3.8 ± 0.6	3.1 ± 0.2

Data are presented as mean ± standard error. Student’s *t-*test was used to compare data between the KLH vehicle and CTGF vaccine group. *P < 0.05.

### Effects of the CTGF vaccine in the UUO mouse model

**Figure 4:**
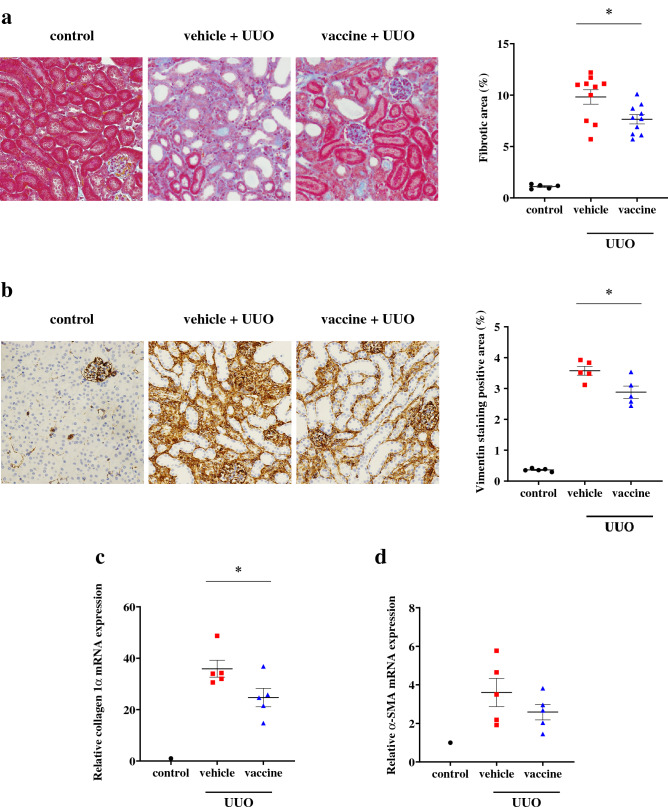
Effects of the CTGF vaccine on renal interstitial fibrosis in the UUO model. Male C57BL/6 J mice were inoculated with the KLH vehicle or CTGF vaccine (*n* = 9/group). Mice were euthanized 1 week after UUO operation. Kidney Sects. (4 µm) were stained with (**a**) Masson’s trichrome and (**b**) vimentin. Ten unselected fields per mouse were captured at × 200 magnification, and stained areas were quantified as percentage of total area. The relative mRNA expressions of (**c**) collagen 1α and (**d**) α-SMA normalized to β-actin were measured with real-time PCR (*n* = 5/group). Error bars show mean ± SEM. Statistical difference between the KLH vehicle and CTGF vaccine group was determined by using Student’s *t*-test. *P < 0.05 as compared with the vehicle.

We also evaluated the effect of the CTGF vaccine on renal interstitial fibrosis in the UUO model. Mice were divided into the non-treated control, KLH vehicle, or CTGF vaccine group at 6 weeks of age (*n* = 5, 9 and 9/group, respectively). At 12 weeks of age (2 weeks after the third inoculation with the vaccine or vehicle), mice in the vehicle and vaccine groups underwent the UUO operation and all mice were euthanized 1 week later (at 13 weeks of age)^28^. Histopathological analyses with Masson’s trichrome-staining showed that the interstitial fibrotic areas were increased after UUO and the expansion of interstitial fibrosis were significantly decreased in mice inoculated with the vaccine compared with the vehicle (7.7 ± 0.4% vs 9.8 ± 0.7%; *P* < 0.05) (Fig. 4a). The increased area of vimentin positive staining was also significantly reduced in the vaccine group compared with the vehicle group (2.9 ± 0.4% vs 3.6 ± 0.3%; *P* < 0.05) (Fig. 4b). The Increased mRNA collagen 1α expression was significantly suppressed and the increased mRNA α-SMA expression tended to be suppressed in the vaccine group compared with the vehicle group (*P* < 0.05 and *P* = 0.26, respectively) (Fig. 4c,d). These results also verified that the CTGF vaccine had nephroprotective anti-fibrotic effects.

### Evaluation of functions of CTGF vaccine-elicited IgG antibodies in vitro

**Figure 5:**
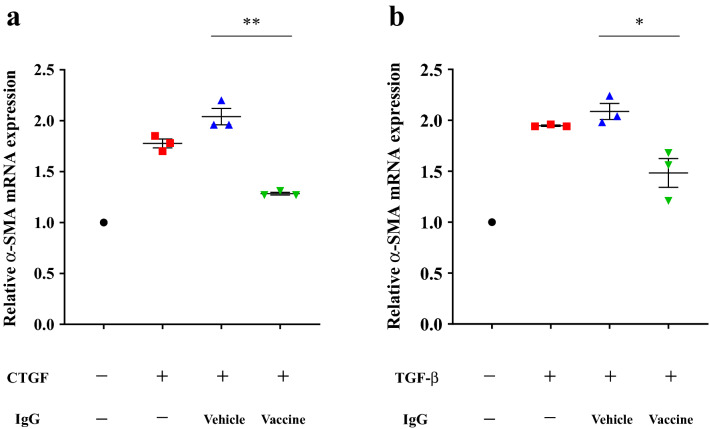
Effects of CTGF vaccine-elicited IgG antibodies on CTGF-induced or TGF-β-induced α-SMA expression in cultured kidney fibroblasts. IgG antibodies were purified from mice inoculated with the KLH vehicle or CTGF vaccine. After pretreatment with the IgG antibodies, NRK-49F kidney fibroblasts were stimulated with rhCTGF. (**a**) The data of relative α-SMA expressions normalized to β-actin expression were obtained by using real-time PCR. (**b**) The effect of IgG antibodies on TGF-β-induced α-SMA expression was examined in the same way. Error bars show mean ± SEM of three independent experiments. Statistical difference between the KLH vehicle and CTGF vaccine group was determined by using Student’s *t*-test. **P < 0.01; *P < 0.05 as compared with IgG from mice treated with the vehicle.

The effects of CTGF vaccine-elicited IgG antibodies on rhCTGF-induced mRNA expression of α-smooth muscle actin (α-SMA) in normal rat kidney fibroblasts (NRK-49F) were examined. NRK-49F cells at approximately 80% confluency in 6-well plates were starved for 24 h. After 2-h pretreatment with or without purified IgGs from mice inoculated with the CTGF vaccine or KLH vehicle, the cells were stimulated with rhCTGF for 24 h. The administration of IgG from mice immunized with the vaccine almost completely suppressed the upregulation of α-SMA, whereas the upregulation was not suppressed by IgG from mice inoculated with the vehicle (Fig. 5a). Next, the effects of CTGF vaccine-elicited IgG antibodies on recombinant human TGF-β-induced mRNA expression of CTGF and α-SMA were also examined in the same manner. In this experimental system, the administration of IgG from mice inoculated with the vaccine, but not the vehicle, significantly suppressed the increased mRNA expression of α-SMA induced by TGF-β (Fig. 5b). TGF-β also increased CTGF mRNA expression, but the CTGF vaccine-induced IgG antibodies had no apparent inhibitory effect on its increased expression (Supplementary Fig. S1). These results indicate that the CTGF vaccine-elicited IgG antibodies had functions of neutralizing the profibrotic actions of CTGF and TGF-β. As for the latter, it is suggested that the vaccine-induced IgG antibodies exerted their antifibrotic actions by at least partially neutralizing the effects of CTGF after its production enhanced by TGF-β, rather than by suppressing TGF-β-induced CTGF expression itself.

### Safety assessment of the CTGF vaccine in the wound healing model

**Figure 6:**
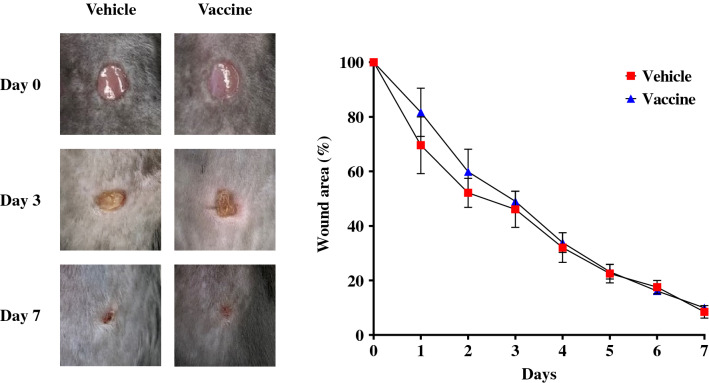
Safety assessment of the CTGF vaccine in the wound healing model. Male C57BL/6 J mice were inoculated with the KLH vehicle or CTGF vaccine (*n* = 5/group). Mice received full-thickness excisional wounds of 5 mm in diameter on their back area. Subsequently, the wound areas were measured daily for 7 days, and the changes in wound area were expressed as the percentage of the original wound areas over time. Error bars show mean ± SEM. Statistical difference between the KLH vehicle and CTGF vaccine groups was determined by using Student’s t-test at each time point.

Because CTGF has a physiological role in wound repair, we used the wound healing model to investigate the impact of the CTGF vaccine on wound repair. Mice were divided into the KLH vehicle or CTGF vaccine group at 6 weeks of age (*n* = 5/group). In the wound healing model mice, immunization with the CTGF vaccine induced antigen-specific serum IgG antibodies comparable to those in the adenine-induced CKD model mice (reciprocal log_2_ titer 14.8 ± 0.4 vs 14.2 ± 1.3; *P* = 0.36). At 16 weeks of age (6 weeks after the third inoculation with the vaccine or vehicle), full-thickness excisional wounds were created, and subsequently the wound areas were measured for 7 days^29^. There were no significant differences in the proportion of wound healing between the two groups at any time point (Fig. 6). This result indicates that CTGF vaccine did not have a significant negative effect on normal wound healing.

## Discussion

In the present study, we developed a peptide vaccine against CTGF to attenuate the development of renal fibrosis. Immunization with this CTGF vaccine elicited IgG antibodies specifically binding to rhCTGF and attenuated the development of renal fibrosis and dysfunction in two mouse models: adenine-induced CKD and UUO models. In an in vitro study, serum IgG antibodies induced by the anti-CTGF vaccination efficiently suppressed CTGF-induced and TGF-β-induced fibrogenesis.

Vaccination appears to be a promising treatment strategy for CKD. Although CKD is recognized as a leading global public health concern from the view point of mortality and medical costs, the treatment options for CKD are still currently limited^1, 6, 7^. Also, non-adherence to medication is highly prevalent among CKD patients for various reasons including less specific symptoms, a need for long-term treatment, and polypharmacy^23^. The development of new therapies for CKD with the potential to achieve high adherence will likely lead to reduced incidences of death, reduced cardiovascular complications and ESRD, and reduced healthcare costs. Considering that vaccines do not require frequent administration owing to their prolonged effect and are cost-effective, vaccination may be an effective approach to address poor drug adherence among CKD patients^24^. In the present study, high antibody titers were maintained over a long period of time, suggesting that the approach using this newly developed vaccine could be an encouraging preventive option.

Renal fibrosis is the common end point of CKD leading to ESRD, independent of the underlying diseases. Interstitial fibrosis is a highly prevalent histological manifestation of CKD, and the degree of fibrosis correlates more strongly with kidney dysfunction than does glomerular injury^30, 31^. Although TGF-β is generally regarded as a central mediator of renal fibrosis, potent inhibition of TGF-β itself can lead to serious adverse events because of its many biological responses including regulation of immune systems and tumorigenesis as well as tissue fibrosis^8–13^. Because vaccine therapy having a long-lasting treatment effect requires a very high level of safety, TGF-β may not be an appropriate target for a vaccine. In this context, CTGF is an attractive candidate target to suppress the development of fibrosis. CTGF is an important modulator of profibrotic TGF-β and antifibrotic BMP activities^14, 25^. Increased expression of CTGF in the kidneys has been observed in various clinical and pre-clinical models, including models of diabetic nephropathy, hypertensive nephrosclerosis, crescentic glomerulonephritis, and chronic allograft nephropathy^15–20, 32^. Plasma and urinary CTGF levels are significantly negatively correlated with estimated glomerular filtration rate (eGFR) in humans, and plasma CTGF levels are reported to be positively correlated with the rate of decline in GFR in patients with type-1 diabetic mellitus and glomerular diseases^33–35^. Suppression of CTGF activity appears to have a relatively high safety, but little is known about the physiological functions of CTGF under non-pathological conditions in the adult organism. Conditional systemic CTGF-knockout mice in the postnatal stage exhibit a healthy appearance with normal histology of kidneys, heart, liver, and lungs, although conventional systemic CTGF-knockout mice die immediately after birth because of thoracic skeletal deformities and pulmonary malformations^19, 36^. Although CTGF has been reported to be partially involved in early wound repair^37^, the CTGF vaccine had no significant negative impact on normal wound healing. Indeed, the anti-CTGF monoclonal antibody FG-3019 has been used in long-term clinical trials for several diseases, and no serious adverse events have been observed^21, 22^. These findings imply that long-lasting inhibition of CTGF by vaccines is likely to be tolerated.

We selected the sequence of amino acids 150–156 of CTGF (GenPept accession no. NP_001892.1) as the vaccine antigen. CTGF, a secreted 36–38 kDa protein, is composed of four distinct domains: insulin-like growth factor-binding domain (domain 1), von Willebrand factor type C repeat domain (domain 2), thrombospondin type 1 repeat (domain 3), and a cysteine knot domain (domain 4). The hinge region between domain 2 and domain 3 is sensitive to proteolytic cleavage. The amino-terminal fragment of CTGF consisting of domains 1 and 2 is predominantly detected in plasma, and the plasma levels of this fragment, but not full-length CTGF or the carboxyl terminal fragment of CTGF, correlate with the severity of fibrotic disease^33, 35, 38^. In particular, domain 2 of CTGF plays a crucial role in fibrosis. Although CTGF is a downstream mediator of profibrotic TGF-β activity, it also directly binds to TGF-β at domain 2, resulting in the enhanced binding of TGF-β to its receptor; furthermore, CTGF interacts with BMPs at domain 2 to inhibit the signaling of BMPs and suppress their renoprotective effect^25^. Recently, domain 4 of CTGF has been reported to promote the fibrogenic pathway through direct interaction with LRP-6 (low-density lipoprotein receptor protein-6)^39^. Although we also considered the binding site of LRP-6 as a candidate for the vaccine antigen, domain 4 of CTGF appears to be less feasible as the vaccine antigen compared with domain 2, because of the lack of evidence in clinical studies at the present time. Therefore, we selected the epitope sequence out of domain 2 (amino acids 150–156), which is a part of the sequence (amino acids 135–157) recognized by the anti-CTGF monoclonal antibody FG-3019^40^. Indeed, our western blotting analyses demonstrated that the CTGF-vaccine-induced serum IgG antibody reacted with the epitope antigen as well as whole CTGF protein (Fig. 1c). In Fig. 1c, a band for recombinant whole CTGF was identified at approximately 50 kDa instead of the original molecular weight of CTGF, 36–38 kDa. This discrepancy might be due to the fact that this recombinant protein may not migrate at the predicted molecular weight of the native molecule, as described on the manufacturer’s website.

In the present study, immunization with the CTGF vaccine significantly reduced the renal interstitial fibrosis area (by 30.9% in the adenine-induced CKD model and 21.4% in the UUO model) compared with vehicle. This is consistent with previous studies that indicated that in the UUO mouse model the antisense nucleotide directed to CTGF reduced the area of renal interstitial fibrosis by approximately 40% and gene knockout reduced the area by 60%^15, 16^, although it should be acknowledged that the efficiency of the CTGF vaccination appears to be less than that of CTGF reduction by antisense nucleotide or gene knockout. On the other hand, administration every second day of FG-3019, the monoclonal antibody targeting domain 2 of CTGF, had almost the same effect on renal fibrosis in the UUO mouse model as our CTGF vaccine^40^. Immunization with the CTGF vaccine also suppressed the elevation of serum creatinine and UN levels, and urine albumin to creatinine ratio was also improved by 17.6%, 25.3%, and 43.4%, respectively, in the adenine-induced CKD model. These results demonstrated that the CTGF vaccine can attenuate not only the development of renal fibrosis but also the decline in renal function. In a previous study, blockade of CTGF by antisense nucleotide reduced urine albumin excretion in a streptozotocin-induced type 1 diabetes mellitus nephropathy mouse model by approximately 60%, whereas the anti-CTGF antibody reduced urine albumin excretion by 20%^17, 18^. These findings also imply that in the treatment of renal fibrosis and CKD, inhibition of domain 2 of CTGF by antibodies is less effective than genetic interventions. There are two possible reasons for this mild vaccine efficacy: (1) the vaccine-elicited antibodies do not directly inhibit the profibrotic functions of domain 4, and (2) the antibodies could not efficiently penetrate the renal interstitium^20^. Furthermore, we should acknowledge that the effects of the CTGF vaccine in advanced CKD models or more severe fibrosis models is unclear. In the adenine-induced CKD model, the final dosing period was set at 3 weeks, albeit for a short period of time, as it was deemed inappropriate to administer adenine to mice for a longer period of time because 3 weeks of adenine administration sometimes resulted in the death in mice or the attainment of the humane endpoint of the experimental animals (rapid weight loss due to renal failure). In fact, it has been reported that hemizygous deletion of CTGF did not improve renal outcome in long-term diabetic nephropathy model, unlike the mild model^41, 42^. Therefore, the effectiveness of the CTGF vaccine may be more limited in advanced CKD models.

Yokoi et al. reported that TGF-β induced extracellular matrix mRNA expression including fibronectin and α1 collagen in cultured renal fibroblasts, and these mRNA expressions were significantly suppressed by CTGF antisense oligonucleotide transfection^43^. TGF-β stimulation also induces production of α-SMA, a marker for myofibroblasts which are regarded as important effector cells of tissue fibrogenesis, in fibroblasts located in various tissues, and the anti-CTGF monoclonal antibody against domain 2 attenuated hyperproduction of it in peritoneal and lung fibroblasts^44–46^. In line with these studies, we showed that the CTGF vaccine-elicited IgG antibodies apparently suppressed TGF-β-induced α-SMA mRNA expression in NRK-49F kidney fibroblasts. In addition, although CTGF itself increases α-SMA production in NRK-49F^47^, in the present study, the vaccine-elicited IgG antibodies almost completely suppressed CTGF-induced α-SMA mRNA expression. These in vitro data clearly showed that the CTGF vaccine attenuated the development of renal fibrosis by inhibiting the production of α-SMA, dependent at least partially on TGF-β signaling.

In conclusion, the CTGF vaccine has the potential to attenuate the development of renal fibrosis and delay the deterioration of renal function with a low frequency of administration. Thus, this CTGF vaccine could be a promising preventive strategy for CKD patients, many of whom have poor medication adherence.

## Methods

### Animals

All animal protocols were approved by The Keio University Institutional Animal Care and Use Committee (Approval No. 18088-1) and were conducted in accordance with the *Institutional Guidelines on Animal Experimentation* at Keio University. Male C57BL/6 J mice were obtained from CREA Japan, Inc. (Tokyo, Japan). All mice were housed with free access to feed (CE-2; CREA Japan, Inc.) and water under standard 12-h light/12-h dark conditions. The study was performed in compliance with the ARRIVE guidelines.

### Preparation of CTGF vaccine and immunization protocols

A mouse CTGF partial peptide in domain 2—a von Willebrand factor type C repeat (GenPept accession no. NP_001892.1; amino acids 150–156, FPRRVKL)—was synthesized for us by Eurofins Genomics (Tokyo, Japan). The peptide was conjugated to KLH using the crosslinker *m*-maleimidobenzoyl-*N*-hydroxysuccinimide ester method (Thermo Fisher Scientific, Inc., Waltham, MA, USA). Before immunization, 20 µg of CTGF–KLH peptide in 100 µL of PBS was mixed with the same volume of Freund’s adjuvant (Sigma-Aldrich Japan, Tokyo, Japan): complete Freund’s adjuvant for the first inoculation, and incomplete Freund’s adjuvant for the subsequent inoculations. Mice in the vaccine group were subcutaneously injected with 20 µg of CTGF–KLH antigen at 6, 8, and 10 weeks of age. Mice in the vehicle group were subcutaneously injected with the same quantity of KLH peptide mixed with adjuvants at the same timing.

### Experiment protocols

In the adenine-induced CKD model, after the third vaccination, mice at 12 weeks of age were fed CE-2 supplemented with 0.2% adenine (Wako, Osaka, Japan) for 3 weeks. Body weight was measured every week from 12 weeks of age. One day before the last day, the mice were housed in individual metabolic cages to measure food and water intake and to collect 24-h urine. Then, after blood samples were collected through cardiac puncture under isoflurane anesthesia, mice were euthanized, and the left kidneys were harvested.

In the UUO model, after the third vaccination, mice at 12 weeks of age were anesthetized with an intraperitoneal injection of a mixture of three types of anesthetic agent consisting of 0.3 mg/kg of medetomidine, 4.0 mg/kg of midazolam, and 5.0 mg/kg of butorphanol^48^. Subsequently, the left ureter was isolated and ligated at two points with a 4–0 silk suture. Mice were euthanized 7 days after the UUO operation, and the left kidneys were harvested.

In the wound healing model, after the third vaccination, mice at 16 weeks of age were anesthetized as described above. Following the removal of the hair on their back area, full-thickness excisional wounds were created with a 5 mm biopsy punch (KAI, corp., Tokyo, Japan). Subsequently, the wound areas were evaluated by measuring the length and width by a ruler daily for 7 days. The changes in the wound area were expressed as the percentage of the original wound areas over time.

### Enzyme-linked immunosorbent assays

Serum antibody titers were determined by ELISA. Microplates (96-well Immulon 1B microplates; Thermo Fisher Scientific, Inc.) were coated with 1.0 µg of CTGF partial peptide (amino acids 150–156) conjugated to bovine serum albumin (BSA) in phosphate buffered saline (PBS) overnight at 4 °C. After the plates were blocked with blocking buffer (PBS containing the non-ionic detergent 0.05% Tween 20 [PBS-T] plus 1% BSA), serum samples from mice were diluted in blocking buffer and added in to each well. After incubation for 2 h at room temperature, and subsequent washing with PBS-T, four classes of goat-anti mouse immunoglobulin—IgG, IgM, IgA, and IgE (SouthernBiotech, Birmingham, AL, USA)—diluted 5000-fold in blocking buffer were added to each well. After incubation for 1.5 h at room temperature and subsequent washing with PBS-T, color was developed by using the TMB Microwell peroxidase substrate system (Kirkegaard & Perry Laboratories, Gaithersburg, MD, USA), and the optical density at 450 nm (OD450) was determined by a microplate reader (Cytation 5; BioTek, Winooski, VT, USA). The endpoint titer was expressed as the reciprocal log of the last dilution that gave an OD450 that was 0.1 units greater than that of the negative control^49^.

### Western blotting analyses

Serum antibodies were evaluated by western blotting analyses for their ability to recognize and bind to recombinant human full-length CTGF protein. Recombinant CTGF protein (9190-CC; R&D Systems, Minneapolis, Minnesota, USA), CTGF partial peptide (amino acids 150–156) conjugated to BSA, and KLH protein were separated by SDS–polyacrylamide gel electrophoresis and blotted onto a polyvinylidene fluoride membrane (Merck Millipore, Darmstadt, Germany). These blots were incubated with serum from mice inoculated with either the vaccine or KLH vehicle. MagicMark XP (LC5602; Thermo Fisher Scientific, Inc.) was used as the protein standard marker. After incubation with HRP-conjugated anti-mouse IgG secondary antibody (Jackson ImmunoResearch Laboratories, Inc., West Grove, PA, USA), the chemiluminescence signal for these blots was detected and analyzed with an ImageQuant LAS 4000 mini chemiluminescence imager (GE Healthcare Bio-Sciences K.K., Tokyo, Japan).

### Biochemistry analyses

Blood and urine samples were collected from adenine-induced CKD model mice at 15 weeks of age. Blood and urine samples were centrifuged, and the levels of serum creatinine, urine creatinine, and serum UN were measured enzymatically. Urine albumin levels were measured by using the Albuwell M kit (Exocell, Inc., Philadelphia, PA, USA) according to the manufacturer’s instructions.

### Histopathological analyses

The extent of interstitial fibrosis in the kidneys of both the UUO and adenine-induced CKD models was histopathologically evaluated. Kidney tissues were fixed in 4% paraformaldehyde and embedded in paraffin. Paraffin Sects. (4 μm) were stained with Masson’s trichrome at Kyodo Byori Inc (Kobe, Japan). To quantitatively evaluate fibroblasts, immunostaining was performed using anti-vimentin antibody (5741 s; Cell Signaling Technology, Danvers, MA, United States) at Morphotechnology Co., Ltd. (Sapporo, Japan). Ten non-overlapping fields at × 200 magnification from each kidney cortex section were randomly selected under a microscope (CX41; Olympus, Tokyo, Japan), and specifically stained interstitial areas (blue color on Masson’s trichrome-staining and brown color on immunostaining for vimentin, respectively) were measured quantitatively by using Photoshop CS4 (Adobe Systems, Inc., San Jose, CA, USA). These results were expressed as the percentage of total imaged area.

### Cell culture and treatments

Serum IgG antibodies were evaluated in an in vitro study for their ability to inhibit the rhCTGF-induced profibrotic signaling pathway. IgGs from mice inoculated with either the vaccine or the KLH vehicle were obtained by using a spin column-based IgG purification kit (Cosmo Bio Co., Ltd., Tokyo, Japan). NRK-49F cells (Japanese Collection of Research Bioresources, Osaka, Japan) were maintained as recommended by the suppliers at 37 °C under 5% CO_2_ in Dulbecco’s modified Eagle’s medium containing 4.5 g/L glucose supplemented with 5% fetal bovine serum and 1% penicillin–streptomycin (Thermo Fisher Scientific, Inc.). For experiments, NRK-49F cells were cultured in a 6-well plate. When the cells reached approximately 80% confluence they were starved in serum-free medium for 24 h. After pretreatment with purified IgGs (10 µg/mL) for 1 h, the cells were stimulated with rhCTGF (50 ng/mL) for 24 h and were then harvested for mRNA extraction. In addition, the effect of purified IgGs on the TGF-β-induced profibrotic signaling pathway was also evaluated in the same manner, by stimulation with recombinant human TGF-β (3 ng/mL) for 24 h (100–21; PeproTech, Cranbury, NJ, USA).

### RNA extraction and real-time polymerase chain reaction (PCR)

Total RNA in kidney and cultured NRK-49F cell homogenates was extracted by using Isogen RNA extraction reagent (Nippon Gene Co., Ltd., Tokyo, Japan) according to the manufacturer’s instructions, and cDNA was prepared by reverse-transcription using PrimeScript RT Master Mix (Takara Bio, Inc., Otsu, Japan). Subsequently, real-time PCR was performed with SYBR Green Master Mix (Thermo Fisher Scientific, Inc.) by using a StepOne device from Applied Biosystems (Thermo Fisher Scientific, Inc.), and the PCR data were normalized to β-actin gene expression with the comparative Ct quantitation method. The primer sequences are shown in Supplementary Table S1.

### Statistical analyses

Data are expressed as mean ± standard error (SEM). Sample size in this animal studies was determined based on our preliminary experiments, which was comparable with previous studies using the similar models. For animal experiments, the number included is described in the figure legend. Each in vitro experiment was replicated at least three times to ensure repeatability of the data. The differences between the KLH vehicle and CTGF vaccine group were analyzed using Student’s *t*-test with SPSS version 27.0 (IBM Inc., Armonk, NY, USA). A *P*-value < 0.05 was considered statistically significant.

## Supplementary Information

Supplementary Information 1.

Supplementary Information 2.

## Data Availability

All data are available in the main text or Supplementary materials. Any remaining information can be obtained from the corresponding author upon reasonable request.
