# Supplementary figures and images for: Vaccination against connective tissue growth factor attenuates the development of renal fibrosis
Source: Sci Rep. 2022 Jun 29;12:10933. doi: 10.1038/s41598-022-15118-5 (PMC9243061; doi:10.1038/s41598-022-15118-5)

## Slide 1
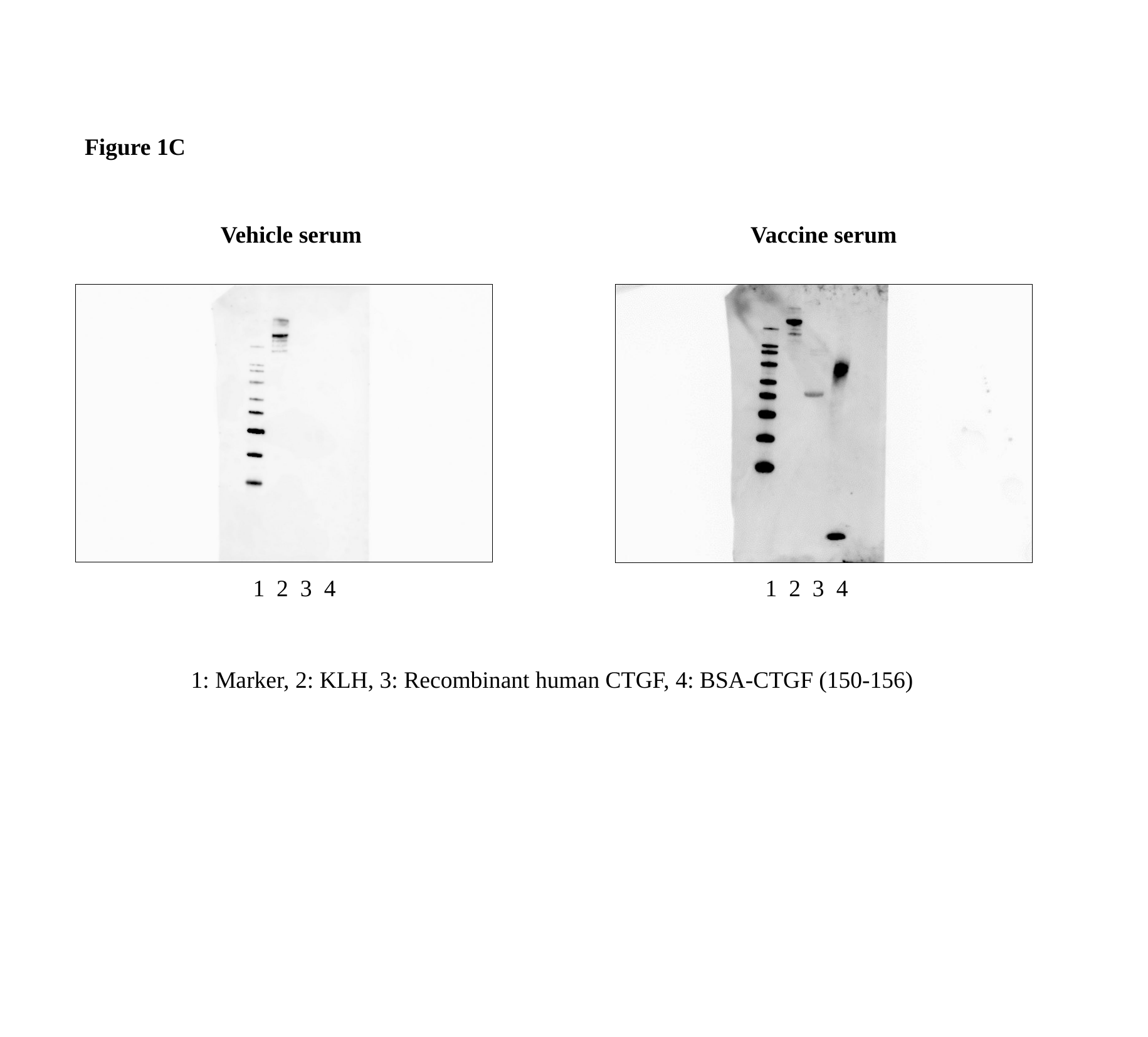

Figure 1C
Vehicle serum
Vaccine serum
 1 2 3 4
 1 2 3 4
1: Marker, 2: KLH, 3: Recombinant human CTGF, 4: BSA-CTGF (150-156)

Supplement: Supplementary file 1 — Supplementary Information 1. [file 41598_2022_15118_MOESM1_ESM.pptx]
