# Supplementary material for: Vaccination against connective tissue growth factor attenuates the development of renal fibrosis
Source: Sci Rep. 2022 Jun 29;12:10933. doi: 10.1038/s41598-022-15118-5 (PMC9243061; doi:10.1038/s41598-022-15118-5)
Supplement: Supplementary file 2 — Supplementary Information 2. [file 41598_2022_15118_MOESM2_ESM.docx]

**Supplementary materials**

**Vaccination against connective tissue growth factor attenuates the development of renal fibrosis**

Takashin Nakayama^1,^ Tatsuhiko Azegami^1,2*^, Kaori Hayashi^1^, Akihito Hishikawa^1^, Norifumi Yoshimoto^1^, Ran Nakamichi^1^, Erina Sugita^1^ & Hiroshi Itoh^1^

**Supplementary Fig. S1. Effects of CTGF vaccine-elicited IgG antibodies on TGF-β-induced CTGF expression in cultured kidney fibroblasts.**

IgG antibodies were purified from mice inoculated with the KLH vehicle or CTGF vaccine. After pretreatment with the IgG antibodies, NRK-49F kidney fibroblasts were stimulated with TGF-β. The data of relative CTGF expressions normalized to β-actin expression were obtained by using real-time PCR. Error bars show mean ± SEM of three independent experiments


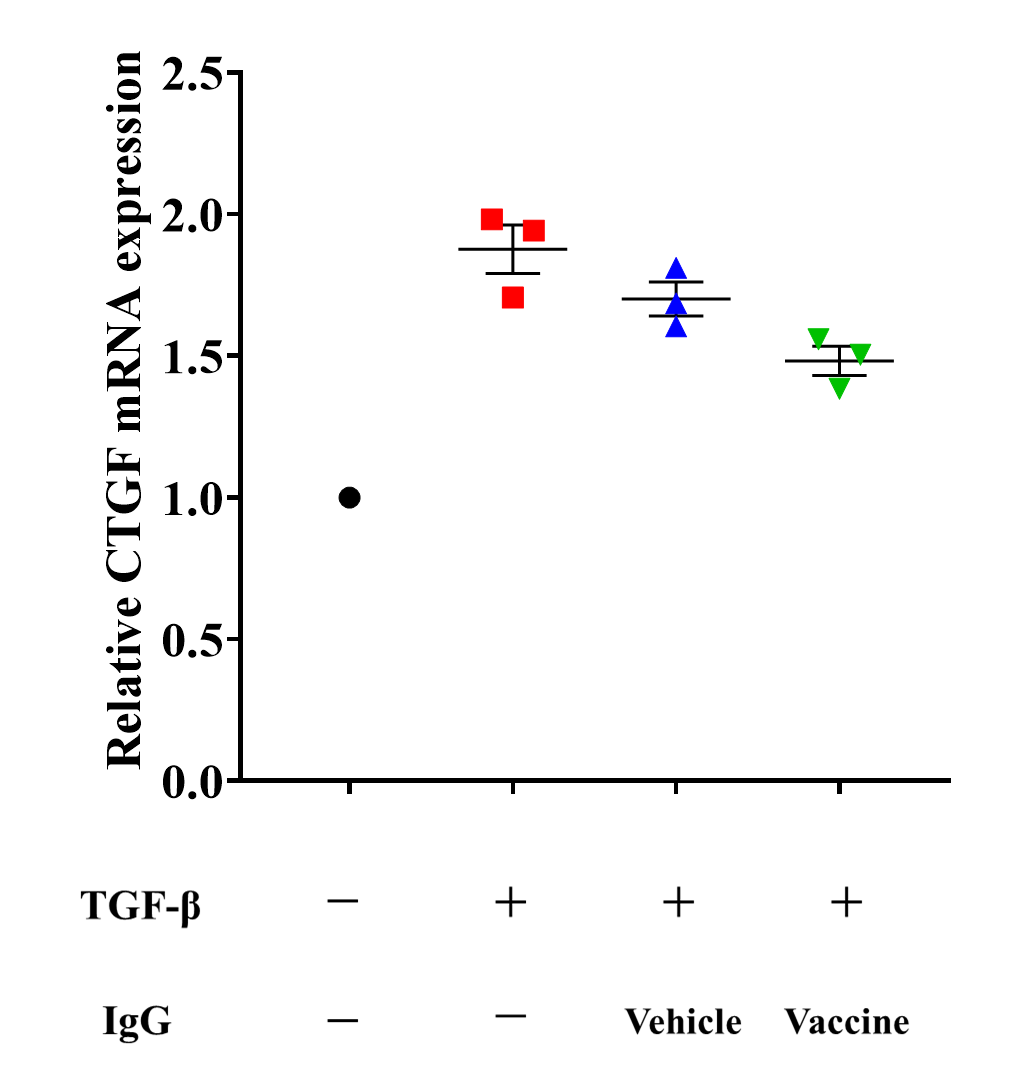


Supplementary Table S1. Primer sequences used for PCR in this study

| Gene | Accession number | Position | Forward primer | Reverse primer |
| --- | --- | --- | --- | --- |
| collagen 1α (Mouse) | NM_007742.4 | 144–253 | ACATGTTCAGCTTTGTGGACC | TAGGCCATTGTGTATGCAGC |
| α-SMA (Mouse) | NM_007392.3 | 346–446 | ACTGGGACGACATGGAAAAG | GTTCAGTGGTGCCTCTGTCA |
| β-actin (Mouse) | NM_007393.5 | 721–909 | TGAGAGGGAAATCGTGCGTGAC | AAGAAGGAAGGCTGGAAAAGAG |
| α-SMA (Rat) | NM_031004.2 | 258–351 | GACCCTGAAGTATCCGATAGAACA | CACGCGAAGCTCGTTATAGAAG |
| CTGF (Rat) | NM_022266.2 | 782–851 | TGGCCCTGACCCAACTATGA | CTTAGAACAGGCGCTCCACTCT |
| β-actin (Rat) | NM_031144.3 | 686–835 | CAGCTGAGAGGGAAATCGTG | CATTGCCGATAGTGATGACC |
